# Stereotactic body radiation therapy (SBRT) for lung malignancies: preliminary toxicity results using a flattening filter-free linear accelerator operating at 2400 monitor units per minute

**DOI:** 10.1186/1748-717X-8-273

**Published:** 2013-11-20

**Authors:** Brendan M Prendergast, Michael C Dobelbower, James A Bonner, Richard A Popple, Craig J Baden, Douglas J Minnich, Robert J Cerfolio, Sharon A Spencer, John B Fiveash

**Affiliations:** 1 Space Coast Cancer Center, Titusville, FL, USA; 2 Department of Radiation Oncology, University of Alabama at Birmingham, Birmingham, AL, USA; 3 Division of Cardiothoracic Surgery, Department of Surgery, University of Alabama at Birmingham, Birmingham, AL, USA

**Keywords:** SBRT, Lung cancer, Toxicity, Flattening filter-free (FFF)

## Abstract

**Background:**

Flattening filter-free (FFF) linear accelerators (linacs) are capable of delivering dose rates more than 4-times higher than conventional linacs during SBRT treatments, causing some to speculate whether the higher dose rate leads to increased toxicity owing to radiobiological dose rate effects. Despite wide clinical use of this emerging technology, clinical toxicity data for FFF SBRT are lacking. In this retrospective study, we report the acute and late toxicities observed in our lung radiosurgery experience using a FFF linac operating at 2400 MU/min.

**Methods:**

We reviewed all flattening filter-free (FFF) lung SBRT cases treated at our institution from August 2010 through July 2012. Patients were eligible for inclusion if they had at least one clinical assessment at least 30 days following SBRT. Pulmonary, cardiac, dermatologic, neurologic, and gastrointestinal treatment related toxicities were scored according to CTCAE version 4.0. Toxicity observed within 90 days of SBRT was categorized as acute, whereas toxicity observed more than 90 days from SBRT was categorized as late. Factors thought to influence risk of toxicity were examined to assess relationship to grade > =2 toxicity.

**Results:**

Sixty-four patients with >30 day follow up were eligible for inclusion. All patients were treated using 10 MV unflattened photons beams with intensity modulated radiation therapy (IMRT) inverse planning. Median SBRT dose was 48 Gy in 4 fractions (range: 30–60 Gy in 3–5 fractions). Six patients (9%) experienced > = grade 2 acute pulmonary toxicity; no non-pulmonary acute toxicities were observed. In a subset of 49 patients with greater than 90 day follow up (median 11.5 months), 11 pulmonary and three nerve related grade > =2 late toxicities were recorded. Pulmonary toxicities comprised six grade 2, three grade 3, and one each grade 4 and 5 events. Nerve related events were rare and included two cases of grade 2 chest wall pain and one grade 3 brachial plexopathy which spontaneously resolved. No grade > =2 late gastrointestinal, skin, or cardiac toxicities were observed. Tumor size, biologically effective dose (BED_10_, assuming α/β of 10), and tumor location (central vs peripheral) were not significantly associated with grade > =2 toxicity.

**Conclusions:**

In this early clinical experience, lung SBRT using a FFF linac operating at 2400 MU/min yields minimal acute toxicity. Preliminary results of late treatment related toxicity suggest reasonable rates of grade > =2 toxicities. Further assessment of late effects and confirmation of the clinical efficacy of FFF SBRT is warranted.

## Background

Stereotactic Body Radiation Therapy (SBRT) involves precise delivery of ablative radiation doses to localized malignancies using concomitant image guidance and/or target tracking. Based on successful early clinical results from phase I and II clinical trials, SBRT has become a common treatment strategy for small primary or metastatic lesions in the lung [1-6]. Despite the advantage of a shortened treatment course compared to conventionally fractionated radiotherapy, the lengthy time required to deliver each high dose treatment fraction is undesirable for immobilized patients and supervising physicians alike. More importantly, prolonged treatment sessions may contribute to intra-fraction motion [7] due to patient discomfort in the treatment position, thereby jeopardizing the delicate therapeutic index of high dose radiation treatment.

**Figure 1:**
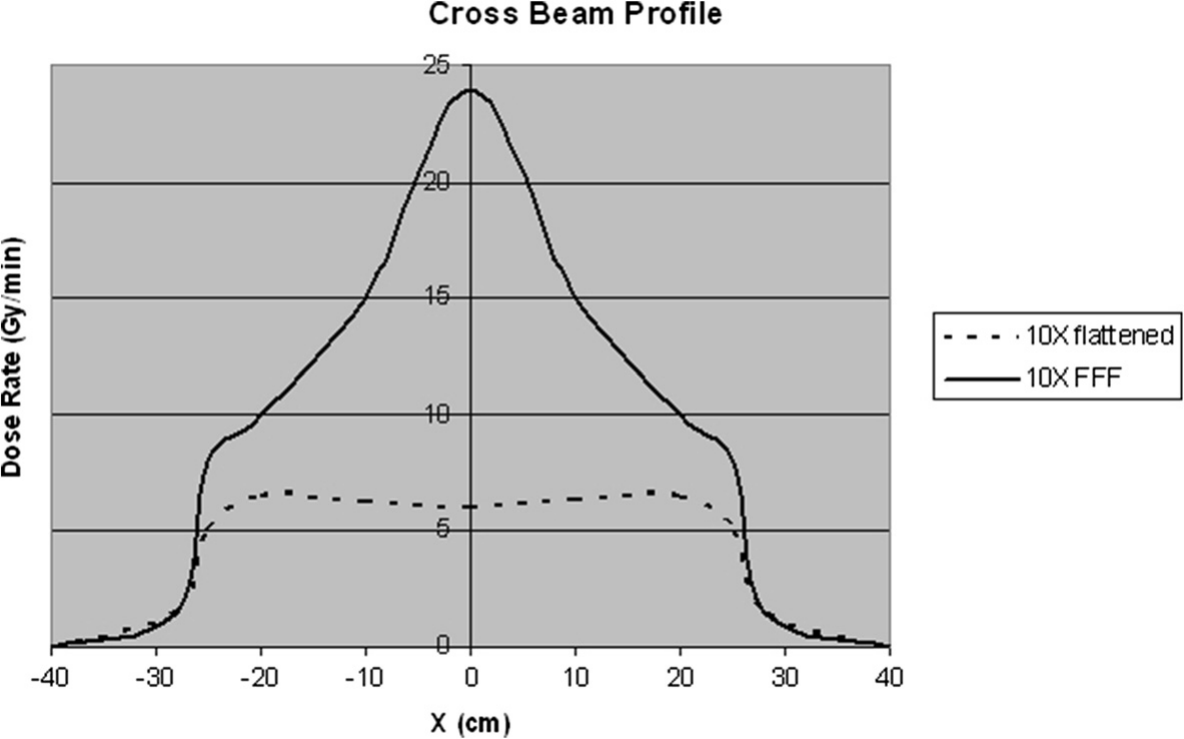
**Cross-beam profile.** Cross beam profile of a conventional 10 megavolt photon beam (dashed line) is compared to the cross beam profile of an unflattened photon beam (solid line) of equivalent energy. The unflattened beam has approximately four times higher dose rate at central axis.

In an effort to shorten SBRT treatment delivery time, manufacturers have developed flattening filter-free (FFF) linacs capable of delivering roughly 4-times higher dose rates compared to conventional linacs with flattened beams (Figure 1). Preclinical data using phantom delivery confirm that FFF linacs can produce dosimetrically equivalent plans [8] while reducing beam-on time by more than 50% [9,10], and early clinical reports confirm these findings [11-14]. However, limited clinical experience with this new technique has led some to question the safety of this approach based on radiobiological principles and clinical experience with high dose rate brachytherapy applications.

The rationale for performing this study was based on knowledge that while DNA damage repair can occur during protracted low dose rate treatment, it may not occur as readily during high dose rate treatments and could yield higher rates of normal tissue toxicity. In order to assess the toxicity with high-dose rate delivery, we retrospectively evaluated the treatment related toxicity resulting from our early lung SBRT experience utilizing a FFF linac operating at a maximal dose rate of 2400 monitor units per minute (MU/min).

## Methods

### Case selection

SBRT has been routinely performed at our institution for lung malignancies since 2008; however, in September 2010 we commissioned a FFF linac (TrueBeam, Varian Medical Systems, Palo Alto) capable of delivering 10MV unflattened photons at a maximal dose rate of 2400 MU/min. Lung SBRT cases performed after September 2010 were almost exclusively performed using this machine, and these consecutively-treated patients formed the basis of this study. For this analysis, SBRT was defined as highly conformal radiotherapy delivered in 3–5 fractions of 6 Gy per fraction or higher with concomitant image guidance and/or target motion tracking.

### Treatment details

All patients were immobilized in the supine position with a custom molded cradle (alpha cradle, Smithers Medical Products Inc, North Canton, OH) and an overhead arm device. All patients underwent CT-guided simulation with respiratory motion assessment using 4D-CT; abdominal compression and other compression devices were not used. Treatment planning was done according to national published standards [5,6], however the final dose schedule and treatment geometry were selected at the discretion of the treating oncologist. Dose schedule was influenced by tumor location; based upon excessive toxicity seen in earlier reports [3], tumors within 2 cm of the proximal trachea-bronchial tree were ineligible for 3-fraction regimens. All patients were treated using 10 MV beams with either sliding-window intensity modulated radiation therapy (IMRT) or volumetric modulated arc therapy (VMAT).

Standard pre-treatment image guidance consisting of paired orthogonal KV X-rays matched to bony anatomy followed by cone beam CT matched to soft tissue and bony anatomy was used in all cases. If significant patient motion was seen on surveillance video, the standard pre-treatment image sequence was re-acquired. Intra-fraction imaging to reaffirm target localization was rarely used due to the shortened treatment times with the increased dose rate.

### Data analysis

After institutional review board approval, we reviewed the electronic medical record for all FFF lung SBRT cases treated at our institution from August 2010 through July 2012. Patients were eligible for inclusion if they had at least one clinical assessment at least 30 days following SBRT. Pulmonary, cardiac, dermatologic, neurologic, and gastrointestinal treatment related toxicities were scored according to CTCAE version 4.0 [15]. Toxicity observed within 90 days of SBRT was categorized as acute, whereas toxicity observed more than 90 days from SBRT was categorized as late. BED_10_ was calculated assuming α/β of 10 by the following formula, where n represents number of fractions, d represents dose per fraction:

$${\mathrm{BED}}_{10}=\mathrm{nd}\left(1+d/\alpha /\beta \right)$$

Factors predicted to influence risk of toxicity—including tumor size, location, number of fractions, and BED_10_—were examined to assess relationship to both acute and late grade ≥2 toxicity. Fisher’s exact test was used to assess tumor location (central vs peripheral) and number of fractions (3 vs more than 3), while logistic regression analysis was used to assess the impact of BED_10_ and tumor size as continuous variables on the development of toxicity (STATA v.12.1, StataCorp, College Station, TX).

### Consent

All patients treated at the University of Alabama at Birmingham Department of Radiation Oncology provide informed consent for the collection of imaging and dosimetry data related to treatment. The use of this data for the current study was approved by the University of Alabama at Birmingham Institutional Review Board.

## Results

Sixty-seven patients who underwent FFF SBRT from August 2010 through July 2012 were identified; 3 patients were excluded as they were lost to follow up. Therefore, 64 patients with adequate follow up were included for analysis. Median SBRT dose was 48 Gy in 4 fractions (range: 30–60 Gy in 3–5 fractions). All patients were treated using 10 MV beams with either sliding-window intensity modulated radiation therapy (IMRT) or volumetric modulated arc therapy (VMAT), chosen at the discretion of the treating physician. The majority (73%) of patients in this cohort were deemed medically inoperable by a board certified thoracic surgeon (DM) based on clinical assessment and pulmonary function tests. The remaining patients were either surgically incurable (by virtue of unresectable disease or distant metastases) or refused to consent to surgery. Table 1 summarizes baseline tumor and treatment characteristics.

**Table 1:** Baseline characteristics

	**Number of patients (%)**
**Histology**	
Non-small cell lung cancer	56 (88%)
Other	6 (9%)
No pathology	2 (3%)
**Dose schedule**	
6 Gy × 5 fractions	2 (3%)
8 Gy × 5 fractions	7 (11%)
9 Gy × 5 fractions	1 (1.5%)
10 Gy × 5 fractions	10 (16%)
10.5 Gy × 5 fractions	7 (11%)
12 Gy × 4 fractions	17 (27%)
18 Gy* × 3 fractions	19 (29%)
Other	1 (1.5%)
**Tumor size**	
≤ 3 cm	36 (56%)
>3 cm	28 (44%)
**SBRT indication**	
Refused surgery	7 (11%)
Medically inoperable	47 (73%)
Surgically incurable	10 (16%)
**Location**	
Central	23 (36%
Peripheral	41 (64%)

Baseline characteristics and treatment details for 64 evaluable patients treated with FFF lung SBRT.

*Includes 20 Gy in 3 fractions without heterogeneity correction.

### Acute toxicity results

Grade > =2 acute pulmonary toxicity was observed in six of 64 patients (9%). Although events were mostly grade 2, one grade 3 case of pneumonitis was observed. There were no grade > =2 acute gastrointestinal, cardiac, skin, or nerve-related events recorded (Table 2).

**Table 2:** Treatment related toxicity

	**Acute toxicity (≤ 90 days)**		**Late toxicity (>90 days)**			
	**Grade 2**	**Grade 3**	**Grade 2**	**Grade 3**	**Grade 4**	**Grade 5**
**Pulmonary**						
Pneumonitis	4	1	4	2	1	1
Atelectasis	-	-	1	1	-	-
Effusion	1	-	1	-	-	-
**Nerve-related**						
Brachial plexopathy	-	-	-	1	-	-
chest wall pain	-	-	2	-	-	-

Type and number of toxic events for 64 patients with grade ≥2 acute toxicity (≤90 days) and subset of 49 patients with grade ≥2 late toxicity (>90 days) at median follow up of 11.5 months.

### Late toxicity results

**Figure 2:**
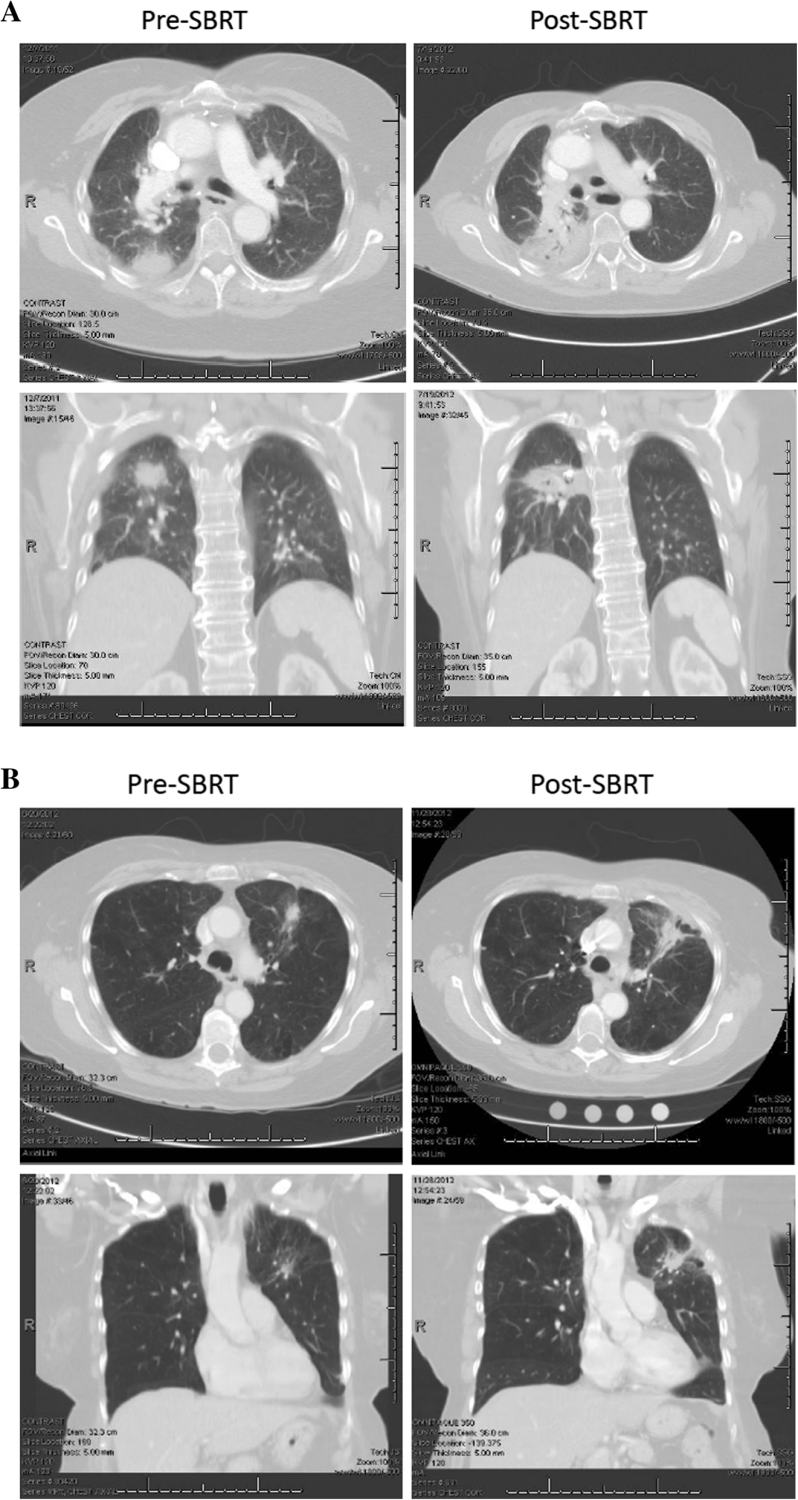
**Radiation associated imaging changes.** Panels **A** and **B** show typical radiation changes that are apparent on chest CT approximately 3–9 months following SBRT. Images on the left show pre-treatment tumor and images on the right show post-SBRT changes. Both axial and coronal planes are provided. The imaging changes depicted above are considered grade 1 radiation pneumonitis by the CTCAE v4.o criteria if they are not associated with clinical symptomatology.

A subset of 49 patients with median follow up of 11.5 months (range 3–25 months) was assessable for late toxicity. Pulmonary toxicities were the most common late events, with asymptomatic grade 1 toxicity based on imaging changes in the irradiated tissues being the most frequently recorded event (20 of 49 patients eligible for late toxicity analysis). Figure 2 displays the characteristic imaging changes frequently observed 3–9 months following pulmonary SBRT and indicate grade 1 toxicity if anaccompanied by clinical symptoms. Grade > =2 late pulmonary events were recorded in 11 pateints (22%). Of note, one grade 5 event (death) occurred in a 75 year old medically inoperable patient with a 4.3 cm peripheral lung tumor treated with 48 Gy in 4 fractions. The patient was hospitalized for two episodes of pneumonia and succumbed to sepsis during the second hospitalization. Although this event was not definitely treatment related, it was included because the inciting infection was localized to the treated lobe. Pre- and post-treatment pulmonary function scores did not indicate clinical deterioration (FEV1 64% and 69% predicted; FVC 78% and 81% predicted, respectively). Overall, non-pulmonary toxic events were rare and limited to three nerve-related late events; there were no grade ≥2 late gastrointestinal, skin, or cardiac toxicities (Table 2).

### Data analysis

Logistical regression analysis of tumor size, number of fractions, BED_10_ (all as continuous variables), and tumor location (central vs peripheral) failed to demonstrate a significant correlation with either acute or late grade > =2 pulmonary toxicity (Table 3). In addition, Fisher’s exact test was used to examine the relationship of tumor location (central vs peripheral) and number of fractions as a dichotomous variable (3 vs >3) with both acute and late grade > =2 pulmonary toxicity. While tumor location did not show an effect (p = 0.66 for acute and p = 0.48 for late toxicity), number of SBRT fractions did suggest that 3 versus 4–5 fractions was associated with lower rates of late pulmonary toxicity (p = 0.04 for late toxicity, p = 0.17 for acute toxicity).

**Table 3:** Statistical analysis

	**Acute toxicity (≤ 90 days)**		**Late toxicity (>90 days)**	
**Clinical factor**	**OR**	**95% CI**	**OR**	**95% CI**
BED_10_	0.98	0.95-1.01	0.98	0.95-1.01
Tumor size	0.97	0.46-2.08	1.47	0.86-2.51
Number of fractions	2.90	0.75-11.2	1.90	0.14-2.27
Tumor location	0.53	0.10-2.85	0.56	0.14-2.27

Four clinical factors predicted to influence risk of pulmonary toxicity were analyzed using logistical regression. BED_10_, tumor size, and number of fractions were analyzed as continuous variables and tumor location was analyzed as a dichotomous variable (Central vs peripheral).

## Discussion

This analysis demonstrates acceptable rates of significant treatment related toxicity following lung SBRT with a FFF linac operating at up to 2400 MU/min. Toxicity observed in this cohort of unselected, consecutively treated patients with lung cancer is comparable to that observed in other published series [2,16,17]. In this experience, acute toxicity at high dose rates is limited to pulmonary events and was mild-moderate in severity.

Although mature data regarding late toxicity is not available, this series provides an early assessment of late treatment related toxicity after FFF SBRT. Grade 2 or greater late pulmonary toxicity was observed in 22% of patients at median follow up of 11.5 months. Severe toxicity (grade 4–5) was uncommon and limited to one death due to pneumonia in a patient with underlying COPD potentially attributable to radiation toxicity and one case where temporary mechanical ventilation was required after pneumonitis symptoms developed in a patient with an existing tracheotomy from prior laryngeal cancer. The majority of observed late events were pulmonary; however, 3 nerve-related events were recorded, including one case of grade 3 brachial plexopathy which resolved after a course of steroids and two other cases of chest wall pain requiring short term narcotics.

On logistic regression analysis factors such as BED_10_, tumor size, number of fractions, and tumor location did not significantly predict for treatment related toxicity. However, when using Fisher’s exact test to examine number of fractions dichotomously, 3 fraction regimens were found to be significantly associated with less late pulmonary toxicity (p = 0.04, two-sided). This interaction was unexpected since 3 fraction regimens deliver a higher BED and therefore would be predicted to cause more toxicity. A likely explanation for these results is that our treatment planning process incorporates the impact of variables such as dose, size, number of fractions and location. For example, lesions would only be planned for 3 fractions if the planner was confident that the size and location were amenable to that approach. Therefore, it is possible that our data reflects the successful integration of previously identified treatment planning goals to prevent known toxicities.

Although use of FFF SBRT is rising, there remains a lack of toxicity data in the literature. In the only other publication specifically addressing toxicity following FFF lung SBRT, Scorsetti et al. report outcomes for 70 patients, 48 of whom had lung tumors [12]. Although separate toxicity was not reported for the lung patients, the overall rate of acute toxicity was 9%, the same rate observed in our experience. Building upon their earlier work, our data includes a preliminary report on late toxic events as well. Late toxic events were more common than acute events, with more than twice as many events observed. Interestingly, not all patients with acute toxicity went on to develop late effects and not all late effects were preceded by acute toxicity, emphasizing the need for longitudinal outcomes studies. Although adequate assessment of late toxicity will require months or years of follow up, it appears to be acceptable at 22% grade > =2 and 10% grade > =3 in our experience.

The question of whether dose rate directly affects cell survival is debatable. Seminal radiobiology studies involving cell lines irradiated *in vitro* established the “dose rate effect,” whereby cell survival decreases as the dose rate increases [18]. Recent preclinical investigations draw conflicting conclusions on the dose rate effect as it relates to FFF linac generated photons. Lohse et al. identified a strong dose rate effect when comparing clonogenic survival in two cell lines irradiated with 400 and 2400 MU/minute [19]. By contrast, Verbakel et al. compared clonogenic survival following irradiation of three different human cancer cell lines with either high dose rate (unflattened) photons or conventional dose rate (flattened) photons and found no difference in cell survival after single or multi-fraction regimens [20]. Sørensen et al. failed to identify a dose rate effect in the range of 5.01- 29.91 Gy/min [21]. Likewise, Ling et al. point out that the dose rate effect is governed by beam-on time, not by instantaneous dose rates, which may be extremely high for a FFF linac [22]. In an earlier study at our institution (involving some of the same patients), we found that actual beam-on time for FFF lung SBRT was on the order of 2–3 minutes [14]. Therefore, assuming fraction doses of 6–18 Gy, the dose rates delivered in this study were 2–9 Gy/min and well within the range examined by Sørensen et al.

In the midst of a basic controversy on whether dose rate affects cell survival, an equally important clinical question remains: does dose rate affect toxicity? Data from this series does not suggest that high dose rate delivery yields elevated treatment related toxicity in lung SBRT. There are likely several explanations for this finding. As previously noted, the absolute dose and dose rates associated with FFF SBRT likely fall into a range where changes are not as significant as those observed in earlier studies using low dose rate experiments. Another sensible explanation for the acceptable toxicity observed is diligent treatment planning with a focus on organ avoidance and gradient index. All patients in this study were treated at an academic medical center from 2010 onward and national treatment planning guidelines aimed at reducing toxicity were adhered to in all cases. Indeed, as demonstrated by Timmerman et al. in a phase II study and echoed in a widely-publicized case report in the New England Journal of Medicine, critical structure avoidance remains a chief concern and determinant of toxicity [2,23].

Despite concerns regarding dose rate and toxicity, FFF treatment carries several promising advantages over conventional flattened photon beam therapy. Preclinical research suggests unflattened beams generate less neutron contamination, lower doses outside the field edge, and less MLC leakage than flattened beams [10,24,25]. Additionally, some have suggested FFF beams could lead to lower rates of secondary malignancies given the observed 70% decrease in scattered photon dose [26]. Some have hypothesized that FFF SBRT will prove to be more efficacious than conventional dose rate therapy owing to the same radiobiological principles discussed earlier. Additionally, it has been shown that FFF SBRT shortens treatment delivery times [13,14], thereby reducing the opportunity for intrafraction motion which could further improve outcomes. Further work will be necessary to determine the clinical significance of these above noted potential advantages for FFF therapy.

Although this is the largest series of toxicity results following lung SBRT with unflattened photons, we recognize several limitations in this study. First, toxicity was scored retrospectively based on clinical documentation and therefore could under- or over-estimate toxicity. Secondly, given the retrospective nature, it is possible that some patients with toxicity did not report back to clinic, which would further contribute to underestimation of toxicity. As previously mentioned, the relatively short follow up of 11.5 months limits interpretation of late toxicity results and ongoing analysis is needed. Lastly, small patient numbers hinder the power of this study to interpret confounding factors that may independently predict toxicity.

## Conclusions

In this early clinical experience, FFF lung SBRT at up to 2400 MU/min yielded acceptable rates of grade ≥2 toxicity. Continued assessment to study the incidence of late effects and further investigation into possible dosimetric correlates of toxicity is warranted.

## Abbreviations

SBRT: Stereotactic body radiation therapy; FFF: Flattening filer free; MU: Monitor unit; IMRT: Intensity modulated radiation therapy; VMAT: Volumetric modulated arc therapy; CT: Computed tomography; KV: Kilovoltage; FEV1: Forced expiratory volume in one second; FVC: Forced vital capacity; MLC: Multi-leaf collimator.

## Competing interests

JF, RP, and MD have received honoraria at CME events sponsored by Varian Medical Systems. The University of Alabama at Birmingham department of radiation oncology has research contracts with Varian Medical Systems.

## Authors’ contributions

BP, JF, MD and RP are responsible for study conception and design. BP is responsible for manuscript writing and assembly of all figures and tables. All authors participated in data collection and interpretation, manuscript revisions, and are in agreement with the content submitted herein.
